# Shiga toxin-producing *Escherichia coli* O26:H11 associated with a cluster of haemolytic uraemic syndrome cases in South Africa, 2017

**DOI:** 10.1099/acmi.0.000061

**Published:** 2019-09-12

**Authors:** Anthony M. Smith, Nomsa P. Tau, Bosco J. Kalule, Mark P. Nicol, Mignon McCulloch, Charlene A. Jacobs, Kerrigan M. McCarthy, Arshad Ismail, Mushal Allam, Jackie Kleynhans

**Affiliations:** ^1^​ Centre for Enteric Diseases, National Institute for Communicable Diseases (NICD), National Health Laboratory Service (NHLS), Johannesburg, South Africa; ^2^​ Faculty of Health Sciences, University of the Witwatersrand, Johannesburg, South Africa; ^3^​ Division of Medical Microbiology, Faculty of Health Sciences, University of Cape Town, Cape Town, South Africa; ^4^​ School of Biomedical Sciences, University of Western Australia, Perth, Australia; ^5^​ Red Cross Children’s Hospital, University of Cape Town, Cape Town, South Africa; ^6^​ Communicable Disease Control, Department of Health, Cape Town, South Africa; ^7^​ Division of Public Health Surveillance and Response, NICD, NHLS, Johannesburg, South Africa; ^8^​ Sequencing Core Facility, NICD, NHLS, Johannesburg, South Africa; ^9^​ South African Field Epidemiology Training Programme, NICD, NHLS, Johannesburg, South Africa

**Keywords:** Shiga toxin-producing *Escherichia coli*, *E. coli*, STEC, EHEC, Shiga toxin, O26, O26:H11, haemolytic uraemic syndrome, HUS, outbreak, cluster, biltong, droëwors, dried meat, dry meat, whole-genome sequencing, WGS, genome, South Africa, Africa

## Abstract

**Introduction:**

Shiga toxin-producing *Escherichia coli* (STEC) are foodborne pathogens that may cause diarrhoeal outbreaks and occasionally are associated with haemolytic-uraemic syndrome (HUS). We report on STEC O26:H11 associated with a cluster of four HUS cases in South Africa in 2017.

**Methodology:**

All case-patients were female and aged 5 years and under. Standard microbiological tests were performed for culture and identification of STEC from specimens (human stool and food samples). Further analysis of genomic DNA extracted from bacterial cultures and specimens included PCR for specific virulence genes, whole-genome sequencing and shotgun metagenomic sequencing.

**Results:**

For 2/4 cases, stool specimens revealed STEC O26:H11 containing *eae*, *stx2a* and *stx2b* virulence genes. All food samples were found to be negative for STEC. No epidemiological links could be established between the HUS cases. Dried meat products were the leading food item suspected to be the vehicle of transmission for these cases, as 3/4 case-patients reported they had eaten this. However, testing of dried meat products could not confirm this.

**Conclusion:**

Since STEC infection does not always lead to severe symptoms, it is possible that many more cases were associated with this cluster and largely went unrecognized.

## Introduction

Shiga toxin-producing *Escherichia coli* (STEC) are primarily foodborne pathogens that may cause diarrhoeal outbreaks and are associated with severe complications, specifically haemolytic-uraemic syndrome (HUS) [1–3]. STEC belonging to serogroup O157 is historically the most commonly described serogroup associated with outbreaks. STEC O157 outbreaks can be linked to a diverse variety of vehicles; recent outbreak reports have been associated with meat products [4–6], vegetables/salads [7–9], dairy/milk products [10] and water [11]. Besides STEC O157, other serogroups of STEC are increasingly reported to be associated with outbreaks. STEC belonging to serogroups O26, O45, O103, O111, O121 and O145 are collectively referred to as the ‘big six’ globally emerging non-O157 STEC [12, 13]. Within the ‘big six’, STEC O26 is frequently detected. Recent reports describing outbreaks involving STEC O26 include a number of different countries. In Israel, an outbreak of STEC O26:H11 affected infants at a nursery in a rural community, where animal contact (animal farming and petting) was determined to be the likely source [14]. In Italy, a community-wide HUS outbreak caused by STEC O26:H11 occurred among children and was associated with the consumption of dairy products [1]. In Romania, an outbreak of HUS caused by STEC O26, occurred in children, where the cause of the outbreak was likely a common food source [15].

In southern Africa, human STEC infections are rarely reported. For this region, very little published data exists concerning the prevalence and epidemiology of STEC infections and HUS cases. The first definitive report of STEC in Africa was reported in 1990 from South Africa and involved a sporadic case of STEC O157:H7 haemorrhagic colitis [16]. The first real notable STEC publication from southern Africa described an outbreak of bloody diarrhoea in Swaziland and the eastern regions of South Africa in 1992, which involved thousands of cases and was caused by STEC O157. In this outbreak, the major contributing factors were carriage of STEC O157 by cattle, death of cattle following drought and heavy rains that resulted in contamination of surface water by dead and dying cattle [17, 18]. In 2011, Smith and coworkers [19] reported on STEC surveillance data for human infections in South Africa for the period 2006 to 2009. Of 2378 diarrhoeal *E. coli* isolates investigated, only 0.6 % (14/2378) were determined to be STEC, with STEC O26 (5/14) and STEC O111 (3/14) most commonly encountered. This low prevalence of STEC in the clinical environment was corroborated by Kalule and coworkers [20], who tested all diarrhoeic stool specimens collected at a South African tertiary referral hospital over a 9 month period, to find STEC in only 5/733 (0.7 %) of specimens tested. Besides clinical cases of STEC in South Africa, there have been occasional reports of STEC from environmental and animal sources. These have included reports of STEC associated with animals for human consumption and their meat products [21–23], vegetables [24–26] and irrigation water [25].

In the present study, we report on STEC O26:H11 associated with a cluster of HUS cases, which occurred in the Western Cape Province of South Africa in 2017. HUS is currently a rarely described syndrome in South Africa.

## Methods

### Identification of HUS cases and the public health response

On 13 February 2017, the Communicable Diseases Control division in the Western Cape Province of South Africa contacted the National Institute for Communicable Diseases (NICD) regarding four cases of HUS. All case-patients had been initially admitted to private hospitals. Case-patients were all female, between the ages of 8 months and 5 years. For all cases, there was no family history to suggest evidence of genetic or atypical HUS. All case-patients initially presented with abdominal cramps, vomiting and diarrhoea (two bloody and two non-bloody), which progressed to HUS 3 to 5 days after initial onset of symptoms. Three of the case-patients were admitted to a children’s hospital with kidney failure, two of whom required renal dialysis. Routine microbiological testing of stool and urine specimens from patients, all tested negative for suspected pathogens, including *Campylobacter* species, diarrhoeagenic *E. coli *, *Salmonella* species, *Shigella* species, *Cryptosporidium* species, Rotavirus and Adenovirus. All cases eventually fully recovered and regained their renal function, and were discharged from hospital off dialysis.

The NICD initiated an outbreak investigation to identify the source and causative agent of the HUS cases. The case definition was as follows: a clinically diagnosed case of HUS in the Western Cape Province of South Africa diagnosed within the period 1 to 28 February 2017.

The investigation occurred over the period 16 February to 13 March 2017. The investigation included visits to all hospitals where the cases presented, to review hospital records to obtain clinical data and interview hospital staff. The homes and workplaces of the direct family members of the cases were visited, for interviews with parents of the cases and general inspection of all premises. Any household member or close contact that experienced diarrhoeal symptoms was also interviewed. All information obtained was captured onto case investigation forms. Data concerning travel history and exposure history were also collected. Where information was obtained about food that was consumed by case-patients prior to the onset of symptoms, then investigators followed up on this information by visiting suppliers of these foods for collection of food samples.

### Extended laboratory testing of stool specimens

Stool specimens collected from all four HUS cases at the initial presentation were sent to a research clinical microbiology laboratory at the Faculty of Health Sciences, University of Cape Town, for extended laboratory testing. By this time, 2 weeks had passed since collection of stool specimens from the patients. Although the specimens had been stored in a refrigerator (4 to 7 °C), the specimens were deteriorated and less than ideal for laboratory testing. Nonetheless, laboratory testing proceeded, briefly described as follows.

Approximately 500 mg of stool was reconstituted into 90 ml of Tryptic Soy Broth (TSB) (Oxoid, Basingstoke, UK) and incubated at 37 °C for 24 h, as per previously described methodology [27]. From these TSB enrichments, a loopful of broth was inoculated onto CHROMagarSTEC agar (CHROMagar Microbiology, Paris, France) for culture and identification of STEC. Suspected colonies of STEC were subcultured on MacConkey agar with crystal violet (Oxoid). Isolates were further identified as *E. coli* using the VITEK-2 automated microbial identification system (bioMérieux, Marcy-l'Étoile, France). Antibiotic susceptibility testing of isolates were performed via the VITEK-2 system using susceptibility-testing cards AST-N255 for Gram-negative bacteria (bioMérieux). TSB enrichments and bacterial cultures were processed to extract total DNA using the MagNA Pure LC Total Nucleic Acid Isolation Kit (Roche Diagnostics, Risch-Rotkreuz, Switzerland) with the MagNA Pure LC automated nucleic acid extraction system (Roche Diagnostics). DNA extractions were tested for the presence of *stx* genes using a LightCycler480 II real-time PCR system (Roche Diagnostics) incorporating the TIB MOLBIOL LightMix Modular Stx2 EHEC Kit (Roche Diagnostics).

### Laboratory testing of food specimens

For testing of food samples, 25 g of the food sample was used as the starting material. The sample was pummeled (ground up) and innoculated into 225 ml of TSB (Oxoid) and incubated at 37 °C for 24 h. Further testing methodology followed exactly as already described above.

### Referral of STEC isolates and DNA extractions to the Centre for Enteric Diseases (CED)

The STEC isolate and DNA extractions from stool specimens, were sent to the CED, NICD for specialized laboratory testing, including all tests further described below. The CED focuses on surveillance and public-health-focused research of pathogens associated with diarrhoea and enteric fevers, and actively assists with the investigation and response to enteric disease outbreaks (including foodborne and waterborne disease outbreaks). The centre provides specialized reference laboratory testing for enteric pathogens.

### Serotyping of the STEC isolate

Serotyping of the STEC isolate was performed by testing of the O-antigen, using the tube agglutination test (antisera manufactured by Statens Serum Institut, Copenhagen, Denmark), using previously described methods [28].

### Analysis of STEC for virulence genes

PCR was used to screen the STEC isolate for the presence of virulence genes associated with the major categories of diarrhoeagenic *E. coli* using previously described methods [29].

### Whole-genome sequencing (WGS) analysis of STEC

Genomic DNA was isolated from bacteria using the Qiagen QIAamp DNA Mini Kit (Qiagen, Hilden, Germany). DNA libraries were prepared using a Nextera XT DNA Library Preparation Kit (Illumina, San Diego, CA, USA), followed by a 2×300 paired-end sequencing runs with 80× coverage using Illumina MiSeq equipment.

Raw data generated on the MiSeq was further analysed using tools available in the CLC Genomics Workbench Software, version 8.5 (CLC bio, Aarhus, Denmark). Using the ‘Trim Sequences Tool’, sequence reads were trimmed to include quality trimming and ambiguity trimming and length trimming to discard reads below a length of 50 bases. Trimmed reads were assembled using the ‘*De novo* Assembly Tool’; the assembly algorithm utilizes de Bruijn graphs to produce contiguous (contig) sequences (minimum contig length was set at 200 bases).

Assembled genome data was analysed using various on-line analysis tools (pipelines) available at the Center for Genomic Epidemiology (CGE) of the Technical University of Denmark (http://www.genomicepidemiology.org/), including the following tools: 'SpeciesFinder', 'PathogenFinder', 'KmerFinder', 'SerotypeFinder', 'VirulenceFinder’, 'Multilocus sequence typing (MLST)' and 'ResFinder'. For investigation of genetic relatedness of STEC isolates, the 'CSI Phylogeny’ tool was used to investigate assembled genome data. The 'CSI Phylogeny' pipeline uses various publicly available programs and the analysis steps, briefly described as follows. Assembled genome data is aligned against a reference genome and single nucleotide polymorphisms (SNPs) are called, filtered and qualified; final qualified SNPs for each genome is concatenated to an alignment; phylogeny is then inferred based on a comparison of SNP alignments of strains. SNP alignments were analysed with iTOL software (http://.itol.embl.de) to generate phylogenetic maximum likelihood trees.

### Analysis of WGS data at the Enterobase platform

Raw WGS data were uploaded and investigated at the Enterobase web-based platform (http://enterobase.warwick.ac.uk/species/index/ecoli). Analysis included a comparison of STEC isolates based on core-genome multilocus sequence typing (cgMLST) data. Phylogenetic cluster analysis of cgMLST data was depicted using a minimum spanning tree.

### Shotgun metagenomic analysis of stool specimens

DNA extractions from stool specimens were enriched for microbial DNA using the NEBNext Microbiome DNA Enrichment Kit (New England Biolabs, Ipswich, MA, USA). Enriched microbial DNA samples were subjected to WGS using Illumina MiSeq next-generation sequencing technology (as already described above), but with a much higher data output of ~1 Gb (~3 million reads) per sample. Raw sequencing data were analysed against microbial databases using the SURPI (sequence-based ultrarapid pathogen identification) pipeline, a computational bioinformatics pipeline for pathogen identification utilizing complex metagenomic sequencing data [30]. Raw sequencing data were further analysed using CLC Genomics Workbench Software (CLC bio) to produce assembled genome data (as already described above). Assembled genome data were analysed using various CGE on-line analysis tools (as already described above), to particularly investigate for the presence and characterization of STEC.

### Data availability

The genome sequence for the STEC O26:H11 strain associated with this cluster of HUS cases has been deposited at NCBI/GeneBank under the accession NGBP00000000 [31].

## Results

### Epidemiological investigation

For all HUS cases, the residential locations and day-care/school locations showed a broad distribution across the district, ranging from 15 to 136 km apart. Parents of the case-patients were asked if they were aware of any recent reports of cases of diarrhoea in their children’s day-care facilities and schools, of which none was reported, so these day-care facilities and schools were not visited. No significant contact with animals was reported in any of the cases. Three of the four cases (cases 1, 3 and 4) were exposed to eating dried meat products, while three of the four cases (cases 1, 2 and 4) regularly consumed fruits. Although some foodstuffs consumed were similar between cases, these were all purchased from different stores and food outlets. Case 3 (the youngest case at 8 months old) had recently started to eat solid foods when symptoms started and at the time was mainly eating baby formula and porridge. However, 3 to 4 days before the onset of symptoms, case 3 also ate dried meat sausage (droëwors) and a meal consisting of rice/pumpkin/mincemeat prepared by caregivers at a day-care facility. Case 4 (2 years old) ate dried meat (biltong) 2 days before showing symptoms; the same dried meat product was eaten by a 2-year-old contact who did not present with any symptoms. Case 1 was also exposed to dried meat products and ate this over 1 week prior to the onset of symptoms.

For cases 2, 3 and 4, similar foodstuffs were eaten by case-patients, family members and close contacts; however no other family members or close contacts experienced diarrhoeal symptoms within the 3 weeks prior to the cases presenting with diarrhoea. In contrast, for case 1, several family members and a housekeeper experienced diarrhoeal symptoms within 3 weeks prior to case 1 presenting with diarrhoea. An uncle, grandmother and grandfather were the first to experience diarrhoeal symptoms, approximately 3 weeks before case 1 presented with diarrhoea. A 2-year-old brother presented with diarrhoea 5 days before case 1 presented with diarrhoea. A housekeeper presented with vomiting and abdominal cramps (limited diarrhoea) 3 days prior to case 1 presenting with diarrhoea.

### STEC culture and PCR diagnosis from stool specimens

For case 4, *E. coli* was cultured from a stool specimen. The culture was identified as STEC O26 and showed the presence of *eae* and *stx2* virulence genes. Antibiotic susceptibility testing showed susceptibility to all the common classes of antibiotics. Analysis of WGS data allowed further characterization of the STEC isolate as follows: serotype O26:H11; MLST sequence type (ST) 21; presence of the *eae*, *stx2a* and *stx2b* virulence genes; presence of the *mdf(A*) gene associated with acquired 'macrolide–lincosamide–streptomycin A' resistance. For case 1, stool specimens were culture negative; however, DNA extractions from the stool tested PCR-positive for *eae* and *stx2* virulence genes, indicating the presence of STEC in the specimen, as Shiga toxin 2 (Stx2) is a marker for the presence of STEC. For cases 2 and 3, stool specimens tested culture-negative and PCR-negative for the *stx2* gene.

### Phylogenetic analysis of the STEC isolate

**Fig. 1.**
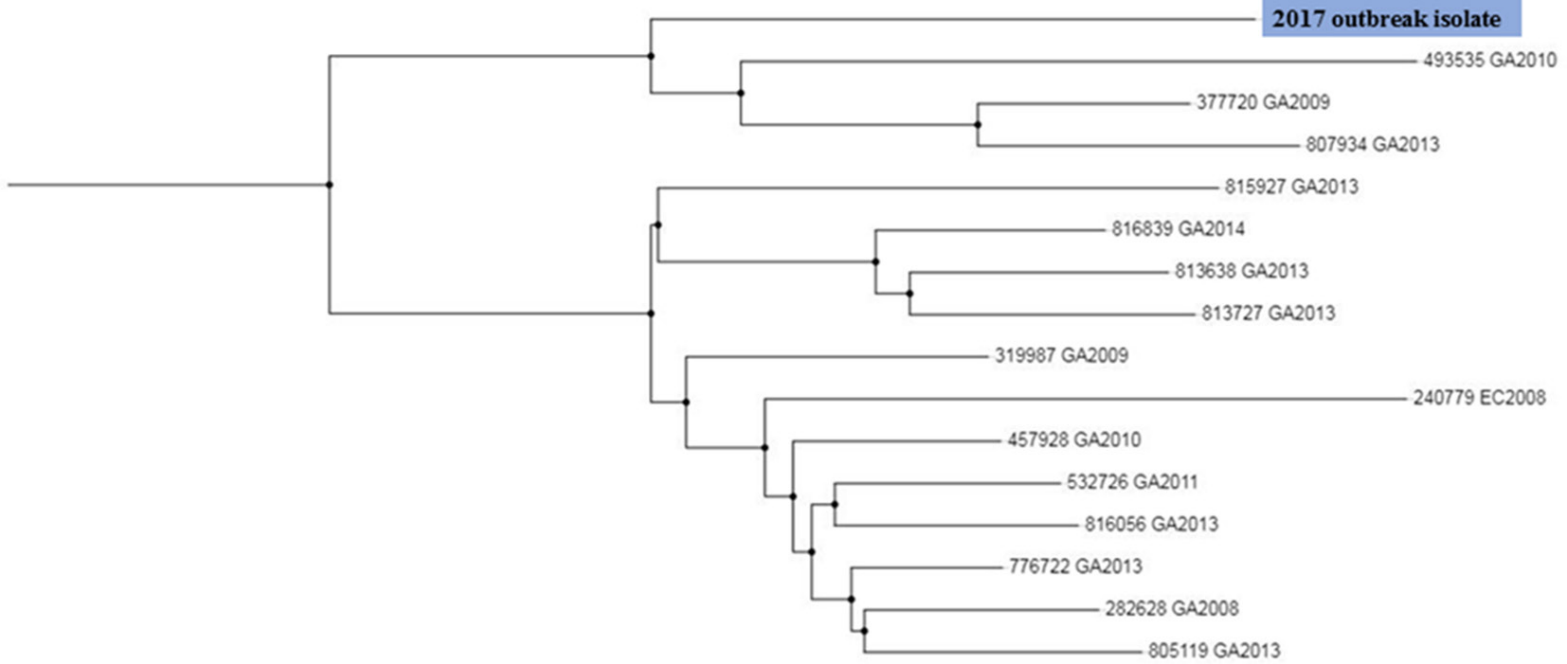
Maximum likelihood tree drawn using SNP alignments from WGS data of STEC O26 isolates recovered in South Africa over the years 2008 to 2017. The current STEC O26:H11 isolate is highlighted.

The CGE 'CSI Phylogeny' pipeline was used to compare the genetic relatedness of our current STEC O26:H11 isolate to that of 15 historical South African STEC O26 isolates recovered over the years 2008 to 2014. A maximum likelihood tree (Fig. 1) drawn using SNP alignments from WGS data, showed that our current STEC O26:H11 isolate was unrelated to all 15 historical STEC O26 isolates, differing by 521 to 771 SNPs as compared to the historical STEC isolates.

**Fig. 2.**
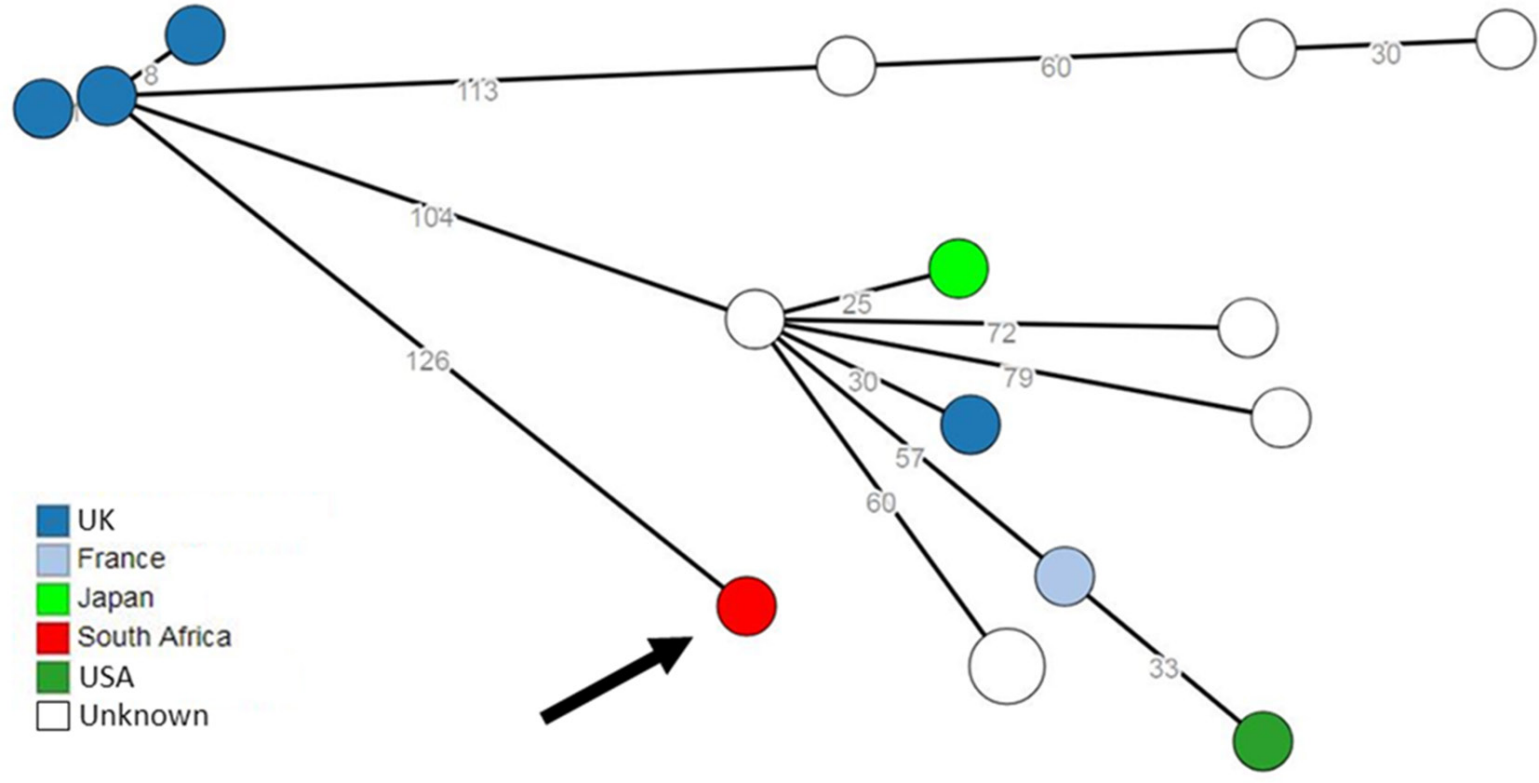
Minimum spanning tree drawn using cgMLST data from STEC isolates in the global EnteroBase database. Circular nodes represent isolate(s) having a unique cgMLST profile. The number values between adjacent nodes indicate the number of allele differences between nodes. Colouring of the nodes represents the country source of the isolates. The current STEC O26:H11 isolate is shown with an arrow.

Comparison to a global collection of STEC O26 isolates was performed at the Enterobase web-based platform. Analysis of cgMLST data showed that the nearest match to our current STEC O26:H11 isolate was a UK isolate recovered in 2016. This nearest match was at 126 allele differences. These data are depicted in Fig. 2, a minimum spanning tree showing the closest isolate matches to our current STEC O26:H11 isolate.

### Shotgun metagenomic analysis of stool specimens

SURPI analysis of the raw shotgun metagenomic data from the stool specimen of case 1, returned an overwhelming match (98 %) to a bacterial DNA database. SURPI analysis within this bacterial database returned an overwhelming and convincing match (96 %) to *E. coli *. SURPI analysis against a viral database found matches to bacteriophages that house *stx2* genes (markers for STEC). Analysis of the assembled metagenomic data at the CGE identified STEC with the identical characteristics as the STEC isolate for case 4, with characteristics as follows: serotype O26:H11; ST21; presence of the *eae*, *stx2a* and *stx2b* virulence genes.

### Laboratory testing of food specimens

Where information was obtained regarding possible food exposures, the suppliers of these foods (different stores and food outlets) were visited for collection of food samples. Some frozen home-prepared meals were also sampled and tested. A variety of food samples were collected and tested. This included dried meat products (beef biltong, kudu biltong, beef droëwors), beef hamburgers, vegetables, cheeses and frozen home-prepared meals. For the food samples tested, results were all culture-negative and PCR-negative for the *stx2* gene.

## Discussion

We investigated a cluster of four HUS cases, which occurred in the Western Cape Province of South Africa in 2017. For two of the cases, STEC O26:H11 was identified from patient stool specimens, confirming that the etiological agent associated with this cluster of HUS cases was STEC O26:H11. The STEC O26:H11 isolate was of subtype ST21 and showed the presence of *eae* and *stx2* virulence genes. This combination of virulence genes is typically associated with severe disease such as haemorrhagic colitis or HUS [32, 33]. Shiga toxin (either Stx1 or Stx2) is a major virulence determinant associated with STEC. In general, Stx2 is usually associated with more severe disease, compared with Stx1. In addition, multiple subtypes of Stx1 and Stx2 can exist, each associated with varying degrees of potency and varying degrees of disease severity. Our current STEC O26:H11 isolate showed the presence Stx2a and Stx2b subtypes. Stx2a, in particular, was also the Shiga toxin identified in the STEC O104:H4 outbreak strain associated with the large outbreak of bloody diarrhoea and HUS that occurred in Germany in 2011 [34]. Studies have shown that Stx2a is among the most potent of all the Shiga toxin subtypes [35].

CED surveillance data for South Africa include only 15 isolates of STEC, recovered over the years 2008 to 2014. Analysis of WGS data of all STEC isolates showed that our current STEC O26:H11 isolate was unrelated to all 15 historical STEC O26 isolates, differing by 521 to 771 SNPs. Our current STEC O26:H11 isolate was also genetically unrelated to all STEC O26 strains in the global Enterobase culture collection. Analysis of cgMLST data for strains within Enterobase showed that the nearest match to our current STEC O26:H11 isolate was a UK isolate recovered in 2016. This nearest match was at 126 allele differences; this many allele differences infer that isolates are unrelated. Therefore, our current STEC O26:H11 isolate has not previously been identified in South Africa, nor does it show any close relationship to STEC isolates described in a global STEC database.

Studies have shown that children under 5 years of age are at greatest risk for developing HUS. This is in agreement with our currently described cluster of cases, where cases were children aged 5 years and under. This has been corroborated by recent reports of STEC O26:H11 HUS outbreaks in several European countries over recent years (2013 to 2015), where children under 5 years of age have primarily been affected [1, 14, 15, 36, 37]. Interestingly, all these STEC outbreak strains showed common phenotypic and genotypic characteristics, which were also characteristic of our current isolate: serotype O26:H11; ST21; presence of *eae* and *stx2a* virulence genes.

We could not establish any epidemiological links among our current four HUS cases. Dried meat products (biltong and/or droëwors) was suspected to be the vehicle of transmission. Three of the four cases reported that they had eaten such dried meat products. However, laboratory testing of dried meat products could not confirm this. Several studies have previously investigated dried meat products (biltong) and found contamination with bacterial pathogens, so biltong certainly can pose a potential health risk [38–40]. Reports have also documented outbreaks of STEC [41–44] and *Salmonella* species [45–48] associated with dried meat products. Biltong, in particular, was associated with an outbreak of *Salmonella enterica* serovar Typhimurium that occurred in London in 2008 [45].

Limitations of this study include the following. At the time of the investigation, HUS was not a notifiable medical condition in South Africa and so investigation was delayed. The NICD was only notified of the cluster of HUS cases 2 weeks after the first diagnosis. As a result, there was a long delay before an outbreak investigation commenced and so stool specimens had deteriorated. In addition, antibiotic therapy of patients had commenced days prior to collection of stool specimens. All of the above factors would have played a role in limiting our ability to detect STEC in all stool specimens by culture or molecular methods. The delayed outbreak investigation led to delayed interviews, so information obtained could have been subject to recall bias. There was also a delay in testing suspected food items, where up to 2 weeks had passed before some food items could be sampled and tested. By this time, the batch of potentially contaminated food could have been replaced with new uncontaminated batches.

In conclusion, we describe a cluster of four HUS cases, possibly associated with consumption of dried meat products. Since dried meat products are a very popular food item among the South African population and since STEC infection does not always lead to severe symptoms, it is possible that many more cases were associated with this cluster and largely went unrecognized. Subsequent to this investigation, HUS was declared a notifiable disease in South Africa in late 2017. We hope this will assist with speedier HUS investigations in the future.
